# Plasticity of the auditory system: theoretical considerations

**DOI:** 10.1590/S1808-86942011000500022

**Published:** 2015-10-22

**Authors:** Vanessa Kappel, Ana Clara de Paula Moreno, Ceres Helena Buss

**Affiliations:** 1 Speech therapist; 2 Speech therapist; 3 Doctoral degree, speech therapist, adjunct professor in the Speech Therapy Department, Santa Maria Federal University (Universidade Federal de Santa Maria)

**Keywords:** hearing, hearing loss, neuronal plasticity

## Abstract

**Abstract:**

Auditory plasticity refers to the possibility of anatomical and/or functional changes in the system where transmission of auditory information takes place. The auditory system is often required in communication; it is important to learn how the auditory system reacts to stimuli in order to improve performance in individual communication of subjects with impaired hearing.

**Aim:**

To review the literature on auditory plasticity and the possibility and ability of plastic responses in the auditory system; also to review the evidence of auditory plasticity.

**Methodology:**

A review of the Brazilian and international literature (journals, books, and graduate studies) was carried out. The MEDLINE, SCIELO, BIREME, PUBMED, and LILACS data bases were consulted, as well as 24 papers from the 1990s to the present date; each paper was assessed for relevance to the topic.

**Conclusion:**

The findings showed that the auditory system is able to reorganize itself if there is variation, whether by by reducing, increasing, or conditioning of sound stimuli. This is evidence of plasticity in the auditory system.

## INTRODUCTION

Neuroplasticity is the ability of the central nervous system to adapt to different stimuli - it is a current object of study. The ability that the auditory system has to change anatomically or functionally is named auditory plasticity^1^.

Hearing loss decreases the ability to perceive sounds, which causes several limitations in individual, social, and emotional development^2^,^3^.

Several studies have investigated the ability of the auditory sensory system to respond with plasticity to different types of injury^4^.

Scientific data on the evidences of plasticity in the auditory system were reviewed because of the importance of hearing, the number of individuals in all age groups being diagnosed with hearing loss, and the relevance of this topic.

A review of the Brazilian and international literature (journals, books, and theses) was made. The following databases were consulted: MEDLINE, SCIELO, BIREME, PUBMED, and LILACS. The key words were: “audition, hearing loss, neuronal plasticity”. As the review progresses, important references cited in the databases above were also consulted. After reading and analysis, this review comprised 24 published papers from the 1990s to the current date, taking into account the relevance of each document for the study topic.

### Hearing

The hearing system consists of auditory sensory organs, nervous system auditory pathways, and brain structures that receive, analyze, and interpret sound information - it is used in communication contexts^5^. It is through language that Man is able to understand the world, organize his universe, make abstractions and convey thoughts and feelings, understand others, interact with the milieu, and acquire knowledge. Thus, the more sound stimuli we receive, the more prepared are we to interact with other individuals^2^,^3^.

However, the auditory system is not always whole. The impact of hearing loss is significant; it affects not only the ability to understand information contained in sound but also the way in which an individual may related with the environment and culture. Additionally, hearing loss may have biological, psychological, and social effects^2^,^6^,^7^.

### Neuroplasticity

“Plastic” derives from the Greek “plastikós”, which means molded. According to the Oxford English Dictionary, plasticity is the ability to undergo a change in shape^1^. The first author to introduce the term plasticity in neuroscience was William James (in The Principles of Psychology, 1890), when referring to the human behavior's inclination to change. James wrote: “Plasticity […] means the possession of a structure weak enough to yield to an influence, but strong enough not to yield all at once. Each relatively stable phase of equilibrium in such a structure is marked by what we may call a new set of habits. Organic matter, especially nervous tissue, seems endowed with a very extraordinary degree of plasticity of this sort; so that we may without hesitation lay down as our first proposition the following, that the phenomena of habit in living beings are due to the plasticity of the organic materials of which their bodies are composed” (p. 68)^1^.

According to Pascual-Leone et al.^1^, plasticity in the brain may be defined as a property of the nervous system to adapt, to change its structural and functional organization. These authors also stated that brain plasticity is an intrinsic property of the nervous system that makes it possible for structure to change in response to experience and changes in the environment.

Behavior and experience equate with the activity of all neurons in the brain. These consist of neuronal networks that are an efficient, spatially compact, and precise medium for processing input signals and generating responses^8^.

Kaas^9^ has stated that there are ordered neuronal connections in sensorial systems in several levels of the brain that link with peripheral neural receptors and form sensory maps. Neuronal activity in a specific site of the somatosensory cortex is evoked by, for instance, stimulating a finger. These maps are altered when the surface of a peripheral receptor changes. If input is removed - such as when a limb is amputated - its somatotropic representation area in the sensory or motor cortex is not silenced; other sensorial receptors will stimulate central neurons that previously responded to the damaged receptors. Thus, plasticity or reorganization of the sensory map has taken place.

Some authors^1^ have argued that plasticity is always active. Therefore, it is a mistake to see plasticity as a property of the brain that is only active following injury to promote functional recovery or compensate functional loss. So, plasticity occurs whenever we undergo new experiences. After brain injury, behavior remains a consequence of whole brain, and thus a consequence of a plastic nervous system.

In the past it was thought that plasticity did not take place in older individuals, and that it was limited in adult nervous systems. However, adult brains have been shown to be able to change and adapt to circumstances. Plasticity is expected to be less efficient with age; however, plastic mechanisms do not end at this or that age, and also take place in the elderly. Thus, it is incorrect to set limits to age groups before beginning treatment^10^, ^11^, ^12^.

During the 1950s, it was stated that language learning was only possible until the age 10 to 12 years; in other words, plasticity was generally associated with critical windows. This was based on the idea that the nervous system had a limited time to develop, and that certain abilities did not develop beyond a specific growth period in children. These ideas are currently being questioned^10^,^13^.

### Plasticity of the Auditory System

Musiek et al.^14^ have defined plasticity of the auditory system as change by nervous cell improvement due to environmental influences.

The hearing system is not fully developed at birth; it matures slowly after the inner ear starts to function. Sensory neuron plasticity is fundamental at the beginning of intrauterine and neonatal life, to consolidate hearing^4^,^15^.

Sensory system plasticity occurs in both peripheral receptor neurosensory tissues and central pathways^4^. Plasticity of the auditory system changes the physiological, biochemical, and/or anatomical properties of central neurons in response to demands for transmitting acoustic information - it is also a biodynamic phenomenon. The auditory system reorganizes itself after any variation in auditory input because stimulus input may be decreased (cochlear injury) or new (postnatal development and when cochlear implants or hearing aids are placed)^16^.

Mecklenburg & Babighian^13^ assessed the results of a longitudinal study at the Medical School of Hannover of adult patients with deafness lasting over 20 years in whom cochlear implants were placed. With regards to auditory plasticity, the authors concluded that age-related neural stability may change, and that plasticity in the auditory nervous center appears to become slower in mature nervous systems, although it persists throughout life.

Musiek & Berge^17^ argue that one of the types of plasticity is developmental. It is present when immature brains start to process sensory information, and remains until adult life. A recent study^18^ has suggested that the human auditory system also has developmental plasticity in the brainstem, and not only the cortex. This discovery has been interpreted in different stimuli and experience contexts.

Changes in the anatomical or physiological properties of the central auditory system may be induced by sensorineural hearing loss (primary plasticity), by reintroducing auditory stimuli (secondary plasticity), or by conditioning^2^,^19^.

### Primary Plasticity

According to Willot^19^, hearing loss induces primary plasticity; however, it is not clear how it affects human hearing. More neurons focus on the frequencies that are still heard when frequency maps are reorganized. This allows the auditory system to continue responding to sound, which benefits hearing. Nevertheless, the responses remain inadequate, as an abnormal number of neurons are excited by specific stimuli, which change the natural neural coding. The sound coding ability is also altered by two other conditions: the system does not realize that several neurons are now responding to the wrong frequencies; and it makes appropriate new associations between stimuli and neural responses.

Interaction plasticity (excitatory or inhibitory) that is evoked by binaural stimuli should also impact perception. This is particularly relevant for deprivation associated with monaural use of hearing aids. An example is the weakened ability of unaided ears - and the strengthened ability of stimulated ears - to evoke central responses. This would lead to unbalanced neural responses where the aided ear would be supported by binaural neurons at the cost of the less stimulated ear. With time, this could result in deprivation. Thus, the term deprivation would not be the most correct word; an association between plasticity and deprivation would cause hearing loss in unaided ears^19^,^20^.

Some authors have given much value to early fitting of hearing aids and balanced stimulation of ears, and have suggested that symmetrical bilateral hearing loss with monaural hearing aid adaptation may become asymmetric because of unequal stimulation^2^,^21^.

### Secondary Plasticity

Studies on the effects of deprivation and auditory stimulation on hearing perception have shown conclusively that the development and function of the auditory system is related with the quantity and quality of auditory input^2^. Rodrigues & Miranda^11^ have shown that adequate stimuli need to be offered to the nervous system so that plasticity mechanisms may be set in motion.

If the central auditory system is able to reorganize itself following injury, the question is whether reintroduction of auditory stimuli by hearing aids or cochlear implants has the potential of provoking auditory plasticity.

Studies on plasticity have suggested that increased auditory stimulation because of amplification may induce secondary plasticity, which facilitates acclimation^19^, ^20^, ^21^.

Munro & Lutman^22^ have suggested that acclimation is the period that follows adaptation to hearing aids; auditory abilities and speech recognition gradually improve because of new cues that become available to users.

If beneficial primary plasticity occurs in an individual, sound amplification may have a negative effect, at least initially, due to distortion between acoustic stimuli and auditory responses that were initially used advantageously by the hearing system. Frequencies that used to go unheard start to be heard again because of hearing aids, and the result is a competition for the old neurons. Thus, neural coding of sounds may raise new problems^19^.

Changes may occur in the organization of the central auditory system between the onset of hearing loss and the moment when hearing aids are used. These should be fitted before the auditory system reorganizes and the opportunity for improving hearing passes. Sound amplification increases auditory stimuli, which may induce secondary plasticity, thereby contributing to aclimation^3^.

The true purpose of hearing aids should be to use stimulus reintroduction to induce secondary plasticity, returning representation maps to the due places. Hearing aids could have two effects if plasticity were used as a clinical tool: to restore sensitivity, and to change the central nervous system^19^.

Mecklenburg & Babighian^13^ have suggested that auditory plasticity is important for the treatment of deaf individuals of all ages in which cochlear implants are placed, as plasticity is related with the ability of the nervous system to adapt to artificial stimuli. These findings demonstrate the capacity of the central nervous system to adapt to new auditory sensations following variable periods of deprivation.

Boéchat^2^ demonstrated plasticity in the auditory nervous system with physiologic evidence in a study that evaluated the effect of deprivation time and stimulation time on auditory sensitivity variations for pure tones and the speech recognition score. The study sample comprised 72 subjects aged from 10 to 86 years and diagnosed with unilateral or bilateral asymmetric sensorineural hearing loss. Forty-three of these individuals were fitted with hearing aids in the worse ear, and 29 did not have hearing aids in the worse ear. Audiologic testing was done (initial, intermediary and final). Variations in sensitivity to pure tones and speech recognition scores were studied among the groups for up to 6 years relative to the stimulation time, duration of deprivation, asymmetry between ears, and level of hearing loss. The author found that hearing aid users had less tone threshold variations and better speech recognition scores, and concluded that there was plasticity due to auditory deprivation in non-users of hearing aids in the worse ear, and secondary plasticity following auditory stimulation in hearing aid users. Furthermore, hearing aid users appeared to gain more within the first two years of introduction of auditory stimuli, while the negative effects of deprivation presented gradually and homogeneously with time. The author concluded that the quantity and quality of stimuli developed and maintained the central auditory system, and also underlined the importance of binaural auditory stimulation.

Amorim & Almeida^21^, on auditory plasticity, studied 16 subjects aged from 17 to 89 years with bilateral symmetrical sensorineural or mixed moderate to severe hearing loss. In one of the steps of this study, 24 ears were analyzed by comparing the mean values of speech recognition scores in each ear at three points in the study: before fitting hearing aids, 4 weeks after, and 16/18 weeks after fitting hearing aids. The authors found that speech recognition values increased in line with the duration of hearing aid use; this improvement may have been due to brain plasticity, but speech recognition score differences were not statistically significant. These authors also related and compared the speech recognition scores among 8 subjects who chose monaural fitting, and compared their mean values with those of hearing aid fitted and non-fitted ears at the three points in the study. The results were not statistically significant, but the findings suggested that with time speech recognition performance improved in aided ears and worsened in non-aided ears. This suggested the existence of acclimation of aided ears and sensory deprivation in non-aided ears. The mean speech recognition scores in right and left ears at the three points of the study were compared in 8 subjects that chose binaural fitting. The results were not statistically significant, but the mean speech recognition scores in both ears increased with amplification, except for the mean speech recognition score of the right ear, in which a decrease was seen after 4 weeks of amplification. The authors^21^ pointed out that although the results were not statistically significant, they concur with the findings of Boéchat^2^.

### Conditioning

Some authors^19^,^23^ have noted that conditioning is also a form of neuroplasticity, and that is fundamental for training auditory abilities. Conditioning is able to change the frequency maps and to generate new connections in response to demands. When correlated with learning, repetition fosters an increase in the number of synapses in related neuronal circuits.

Studies have shown that hearing improved after auditory training. Ambient influences - such as auditory training - stimulated neural structures involved with the performance of trained hearing abilities, thereby benefiting individuals. Neural plasticity, therefore, is essential for improvements gained in auditory training^17^,^24^,^25^.

## CONCLUSION

Professionals involved in habilitation and/or rehabilitation of patients with hearing loss need to understand plasticity in the auditory system, and consider it an important clinical tool. Thus, the possibility of primary, secondary, and/or conditioned plasticity should be taken into account in clinical practice. Future studies will be very helpful to add knowledge about plasticity in the auditory system. Our review of the literature showed that the auditory system is able to reorganize itself when the input of auditory stimuli increases or decreases. So, subjects with hearing loss who require hearing aids or cochlear implants may benefit in their social, emotional, and intellectual aspects, and enjoy an improved quality of life.
